# Lethal and Non-Lethal Functions of Caspases in the DNA Damage Response

**DOI:** 10.3390/cells11121887

**Published:** 2022-06-10

**Authors:** Karla E. Lopez, Lisa Bouchier-Hayes

**Affiliations:** 1 Department of Pediatrics, Division of Hematology-Oncology, Baylor College of Medicine, Houston, TX 77030, USA; karla.lopez@bcm.edu; 2 Department of Molecular and Cellular Biology, Baylor College of Medicine, Houston, TX 77030, USA; 3 William T. Shearer Center for Human Immunobiology, Texas Children’s Hospital, Houston, TX 77030, USA

**Keywords:** caspases, DNA damage, genomic instability, cell cycle, apoptosis

## Abstract

Members of the caspase family are well known for their roles in the initiation and execution of cell death. Due to their function in the removal of damaged cells that could otherwise become malignant, caspases are important players in the DNA damage response (DDR), a network of pathways that prevent genomic instability. However, emerging evidence of caspases positively or negatively impacting the accumulation of DNA damage in the absence of cell death demonstrates that caspases play a role in the DDR that is independent of their role in apoptosis. This review highlights the apoptotic and non-apoptotic roles of caspases in the DDR and how they can impact genomic stability and cancer treatment.

## 1. Introduction

Cells undergo constant DNA damage caused by both exogenous and endogenous sources [1,2]. To overcome this, cells activate a network of cellular pathways known as the DNA damage response (DDR) [3]. When activated, the purpose of these pathways is to prevent the accumulation of damaged cells that could potentially become malignant and promote tumorigenesis. To achieve this, cells activate pathways that repair the damaged DNA, that induce cell cycle arrest to allow for repair, or that eliminate the damaged cells by apoptosis. The activation of the apoptotic pathway is regulated by members of the caspase family [4]. Although the role of caspases in apoptosis has been very well studied to date, their contribution to the DDR has been considered to be limited to the removal of damaged cells. However, it is emerging that caspases also make important contributions to DNA damage and repair pathways by engaging non-apoptotic roles.

## 2. The DNA Damage Response

The DDR includes a complex network of signaling pathways comprising of a variety of DNA repair mechanisms. The pathways that are most prevalently impacted by caspases are those that repair single strand or double strand breaks (SSBs or DSBs) in the DNA and generally converge on the activation of ataxia–telangiectasia mutated (ATM), ataxia–telangiectasia- and Rad3-related (ATR), and DNA-dependent protein kinase catalytic subunit (DNA-PKcs). Here, we aim to discuss the specific contributions of caspases to the DDR and thus provide a brief summary of these main DDR pathways that are impacted by caspases. The full complexities of these pathways have been extensively reviewed elsewhere [5,6]. DSBs arise from both endogenous and exogenous sources, such as free radicals and ionizing radiation, respectively [7,8], as well as V(D)J recombination and class switch recombination (CSR) [9,10]. SSBs can be induced by ionizing radiation, reactive oxygen species (ROS), and as a result of replication errors resulting in single strand DNA (ssDNA) [11,12]. Both types of breaks are detected and bound by polyADP-ribose polymerase 1 (PARP1), which repairs the DNA [13]. PARP1 was one of the earliest caspase substrates identified, and its cleavage serves to inhibit the DNA damage response during apoptosis [14].

Replication stress results from anything that interrupts DNA replication by inducing DNA replication fork stalling. Stimuli that induce replication stress include nucleotide depletion, DNA interstrand cross-links (ICLs), and oncogene activation [15]. ssDNA is recognized and coated by replication protein A (RPA) comprised of RPA70, RPA32, and RPA14 subunits that bind to ssDNA with very high affinity [16]. These RPA-bound-ssDNA stretches recruit ataxia–telangiectasia-mutated and ATR interacting protein (ATRIP). In turn, ATRIP recruits ATR [17]. This complex has a basal level of kinase activity until the DNA repair scaffold protein TopBP1 is bound and activates ATR [18]. TopBP1 also recruits the 9-1-1 complex and RAD17 to the site. The 9-1-1 complex is comprised of RAD9, RAD1 and HUS1, which together form a DNA sliding clamp that is loaded onto DNA by RAD17 [19]. Binding of 9-1-1 stabilizes TopBP1, amplifying the activation of ATR [17,19].

Once activated, ATR phosphorylates downstream substrates, including checkpoint kinase 1 (Chk1), which phosphorylates Cdc25A, inducing its degradation. This prevents Cdc25A from activating Cyclin A/E, triggering the intra S-phase checkpoint [20]. The activation of the intra-S-phase checkpoint prevents the cell cycle from progressing to G2 until repair and DNA replication is complete. Active Chk1 similarly inhibits Cdc25C-mediated activation of Cyclin B activating the G2/M checkpoint, which prevents damaged cells from entering mitosis [21]. Alternatively, under UV irradiation, Chk1 and the kinase polo-like kinase 1 (PLK1) phosphorylate and activate RAD51 to promote homologous recombination and repair [22]. In the presence of ICLs, ATR phosphorylates FANCI [23] and FANCD2 [24] and Chk1 phosphorylates FANCE [25]. This culminates in the activation of the Fanconi anemia pathway, which resolves the ICLs. Chk1 also phosphorylates and activates the tumor suppressor p53 [26], which induces cell cycle arrest by inducing the expression of *CDKN1A* (the gene encoding p21), DNA repair through XPC, and apoptosis by inducing the expression of p53 upregulated modulator of apoptosis (*PUMA*) [27].

If they are not repaired, ssDNA can be converted into DSBs, resulting from collapse of the DNA replication fork [28]. DSBs produced in this way or by exogenous sources can be repaired by two different pathways: homologous recombination (HR) or non-homologous end joining (NHEJ), which are generally regulated by the stage of the cell cycle [29]. During the S-phase of the cycle, DSBs are repaired by HR due to the presence of a sister chromatid that can be used as a template during the repair process. DSBs are detected by the MRE11–RAD50–NBS1 (MRN) signaling complex comprised of the proteins MRE11, RAD50, and NBS1. The MRN complex recruits the kinase ATM to the DNA damage site, resulting in ATM auto-phosphorylation and activation [30].

Once activated, ATM amplifies the DDR by phosphorylating downstream substrates including H2AX, to produce γH2AX [31] which recruits DNA repair proteins to the sites of DSBs. The phosphorylation of H2AX is amplified by mediator of DNA damage checkpoint protein 1 (MDC1), which binds to H2AX and promotes the binding of additional MRN complexes resulting in more ATM activation and an accumulation of DNA repair proteins at the DNA damage sites [32,33]. ATM phosphorylates and activates Chk2 [34], which induces cell cycle arrest by inhibiting Cdc25A and Cdc25C to activate the intra-S and G2 checkpoints, respectively [35,36]. ATM and Chk2 also induce cell cycle arrest by activating p53, since they are both able to phosphorylate p53 [37]. ATM has a direct role in DNA repair by activating breast cancer 1 (BRCA1), which initiates repair by HR [38].

**Figure 1:**
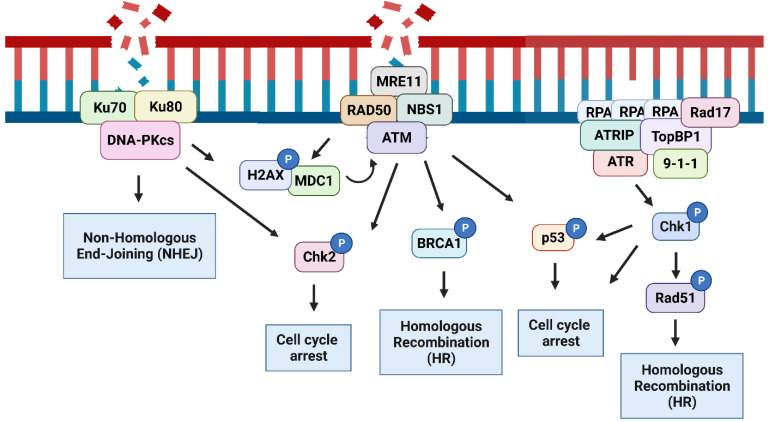
The DNA damage response. Upon DNA damage, a network of pathways known as the DNA damage response (DDR) is activated. Double strand breaks (DSBs) are detected by the Ku70/Ku80 heterodimer or the MRN complex (MRE11, RAD50, and NBS1). These sensors then recruit and activate the apical kinases DNA-dependent protein kinase (DNA-PK) through the Ku70/Ku80 complex or ataxia–telangiectasia-mutated (ATM) through the MRN complex. Activated DNA-PKcs induces DNA repair through non-homologous end-joining (NHEJ). Activated DNA-PKcs can phosphorylate H2AX and checkpoint kinase 2 (Chk2). ATM phosphorylates H2AX, amplifying the DDR by recruiting more DNA repair proteins, such as the mediator of DNA damage checkpoint 1 (MDC1) to the sites of DNA damage; Chk2-inducing cell cycle arrest; and breast cancer 1 (BRCA1)-inducing DNA repair by homologous recombination (HR). In the presence of single strand DNA (ssDNA), replication protein A (RPA) coats the ssDNA, resulting in the recruitment of ATRIP/ataxia–telangiectasia and Rad3-related (ATR) complexes, resulting in ATR activation, which is amplified by the further recruitment of TopBP1, 9-1-1, and Rad17. ATR induces cell cycle arrest through the activation of checkpoint kinase 1 (Chk1) and phosphorylation of p53 and promotes DNA repair by HR through Rad51 activation.

Although active throughout the cell cycle, NHEJ is predominantly activated during the G1 and G2 phases of the cell cycle [29]. NHEJ is triggered by the activation of the serine-threonine kinase DNA-PKcs. The Ku70/Ku80 complex recognizes DNA ends formed as a result of DSBs. This heterodimeric complex moves along the DNA allowing DNA-PKcs to load onto the DNA end. This forms the active DNA-dependent protein kinase holoenzyme (DNA-PK) complex, which dimerizes with the DNA-PK complex that forms on the DNA end at the other side of the break. The DNA-PK complex recruits several NHEJ proteins, including XLF, XRCC4, and DNA ligase IV. These proteins join ligatable ends. If the ends are not ligatable then processing enzymes, such as PNKP, are recruited to remove or repair the damaged DNA prior to ligation [39]. Unlike HR, NHEJ does not require a sister chromatid. For this reason, NHEJ joining is thought to be more prone to errors, but this has been subject to some controversy [40] (Figure 1).

While the activation of these pathways is mainly regulated by the type of DNA lesion, studies have shown a crosstalk between the different DNA repair pathways. For example, the phosphorylation of H2AX by ATM permits recruitment of 53BP1, which then recruits TopBP1 and 9-1-1, resulting in the activation of the ATR pathway [41]. Likewise, upon replication fork stalling, ATM is phosphorylated in an ATR-dependent manner [42]. Crosstalk between ATM, ATR, and DNA-PK to regulate p53 has also been demonstrated. Under normal conditions, RPA binds and sequesters p53 [43]. However, upon DNA damage, the phosphorylation of p53 by ATM and ATR and the phosphorylation of RPA by DNA-PK disrupts the interaction between RPA and p53 to promote DNA repair [44]. Furthermore, in the absence of ATM, ATR and DNA-PKcs have been shown to phosphorylate known ATM substrates, such as H2AX and Chk2 [45,46].

## 3. DNA Damage-Induced Apoptosis

The caspase family is a family of cysteine aspartate proteases primarily known for their role in cell death [4]. The members of this family that are involved in apoptosis are divided into initiator and executioner caspases. Initiator caspases include caspase-2, caspase-8, caspase-9, and caspase-10, and executioner caspases include caspase-3, caspase-6, and caspase-7. The executioner caspases are present in the cell as inactive dimers and are activated by cleavage [47]. They cleave a number of structural and regulatory proteins to bring about the death of the cell [48]. Initiator caspases are present in the cell as monomers that are activated at the apex of their respected pathways by their recruitment to activation platforms [49,50]. This results in the induced proximity of caspase monomers, facilitating dimerization, which is required for activation. Once activated, the caspase undergoes autocleavage, which generally functions to stabilize the active enzyme [50,51]. The main substrates for initiator caspases are the executioner caspases. The exception to this is caspase-2, which activates the executioner caspases indirectly by engaging the intrinsic pathway (see below) [52]. Thus, the primary function of initiator caspases is to directly or indirectly activate the executioner caspases to induce apoptosis (Table 1).

**Figure 2:**
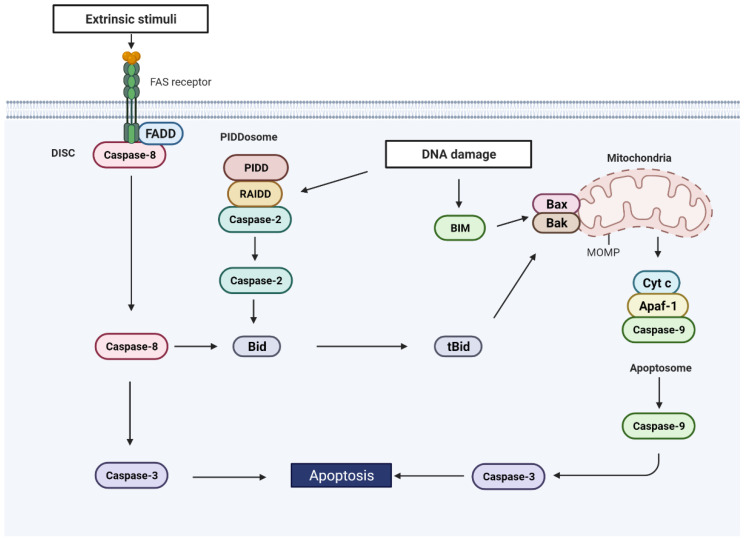
The extrinsic and intrinsic apoptotic pathways. The activation of the extrinsic pathway is initiated by the binding of ligands to receptors located on the plasma membrane. Upon activation of these receptors, the death-inducing signaling complex (DISC), composed of Fas-associated protein with death domain (FADD) and caspase-8, is formed, activating caspase-8. Active caspase-8 then cleaves caspase-3, which cleaves downstream substrates, culminating in apoptosis. DNA damage activates the intrinsic pathway, leading to the activation of BH3-only proteins and allowing the oligomerization of BAX and BAK and the release of cytochrome c (Cyt c) from the mitochondria, following the mitochondria outer membrane permeabilization (MOMP). Cytosolic Cyt c forms a complex with apoptotic peptidase activating factor 1 (APAF-1) and caspase-9 called the apoptosome, resulting in the activation of caspase-9, caspase-3, and apoptosis. DNA damage can also induce PIDDosome assembly, a complex comprised of p53-induced protein with a death domain (PIDD), RIP-associated ICH-1/CAD-3 homologous protein with a death domain (RAIDD), and caspase-2, resulting in caspase-2 activation. Once activated, caspase-2 cleaves the pro-apoptotic protein BID to tBID, inducing MOMP and apoptosis. Alternatively, the activation of caspase-8 by extrinsic signals can engage the activation of the intrinsic pathway by cleavage of BID.

DNA damage generally induces caspase activation and apoptosis by engaging the intrinsic or mitochondrial pathway. This pathway is engaged when members of the B-cell lymphoma 2 (BCL2) protein family of pro- and anti-apoptotic proteins are activated. The BCL2 family includes three subgroups, pro-apoptotic BH3-only proteins (e.g., BID, BIM, and BAD), pro-apoptotic activator proteins (BAX and BAK), and anti-apoptotic proteins (BCL2, BCL-XL, and MCL-1) [69]. DNA damage leads to the activation of BH3-only proteins as well as BAX and BAK. Certain BH3-only proteins (BID and BIM) also activate BAX and BAK, while others inhibit the anti-apoptotic BCL2 family members [70]. Collectively, this permits the oligomerization of BAX and BAK, resulting in mitochondrial outer membrane permeabilization (MOMP) and allowing the release of cytochrome c and other intermembrane space proteins from the mitochondria. Once cytochrome c is released, it forms a complex with apoptotic peptidase activating factor 1 (APAF-1) and caspase-9, known as the apoptosome, resulting in caspase-9 dimerization and activation [71]. Activated caspase-9 then cleaves and activates caspase-3, resulting in apoptosis (Figure 2).

Caspase-2 engages the intrinsic pathway through the cleavage of BID [52]. The stimuli that engage this pathway are generally conditions that lead to DNA damage, such as polyploidy or genotoxic stress. Caspase-2 is activated by the PIDDosome, which is composed of the p53-induced protein with a death domain (PIDD) and the RIP-associated ICH-1/CAD-3 homologous protein with a death domain (RAIDD) [72]. PIDD is a p53 target protein and therefore is upregulated in response to DNA damage [73]. However, PIDD can be expressed in the absence of p53 [74]. In addition, caspase-2 has been shown to be activated by DNA damage to induce apoptosis in the absence of p53, indicating that alternate modes of activation exist. For example, in the absence of p53 and when Chk1 is inhibited, irradiation activates caspase-2 to induce apoptosis [75]. When Chk1 is blocked, irradiation activates ATM, which phosphorylates PIDD1, resulting in RAIDD recruitment, the formation of the PIDDosome, and caspase-2 activation [76]. Similarly, when Chk1 is inhibited, the induction of mitomycin C-induced ICLs induce ATR and ATM-dependent phosphorylation of PIDD1 [77]. These studies suggest that caspase-2 is activated downstream of ATM and ATR in the absence of p53 when Chk1 is repressed.

Caspase-2 is activated by a number of other inducers of DNA damage, including double strand break inducers like the topoisomerase II inhibitor etoposide, irradiation, and the alkylating agent cisplatin as well as SSB inducers like the topoisomerase I inhibitors, camptothecin and topotecan [78,79,80]. However, in each case apoptosis is not completely inhibited in the absence of caspase-2 and is often only inhibited to 50% or less. In addition, the caspase-2 dependence of apoptosis induced by DNA damage is more prevalent in transformed cell lines compared to primary cells, indicating that an oncogenic stress or similar may be required for caspase-2 to induce apoptosis [80]. Thus, additional routes to MOMP by DNA damage inducers exist. This includes the oligomerization of BAX and BAK by the direct binding of BIM or PUMA [81,82]. In addition, p53 has also been shown to directly bind to BAX and induce apoptosis independently of its transcription factor function [60].

The second major pathway to cell death is the extrinsic pathway (Figure 2). This pathway is activated when the signal to initiate apoptosis is received from outside the cell in the form of death ligands. The extracellular ligands, CD-95L/FasL, TNF-related apoptosis-inducing ligand (TRAIL), and tumor necrosis factor (TNF) bind to their cognate plasma membrane bound death receptors CD-95/Fas, TRAILR-1 and -2, and TNF receptor 1 (TNFR1), respectively. The activation of CD95 and TRAIL results in the recruitment of Fas-associated protein with death domain (FADD) and caspase-8, forming the death-inducing signaling complex (DISC) and resulting in caspase-8-induced dimerization, activation, and subsequent executioner caspase activation [83]. DNA damage can engage this pathway through upregulation of *CD95* and *CD95L* expression. The *CD95* promoter region contains p53 responsive elements and is upregulated by the wild-type but not mutant p53 in many cancer cell lines [61]. Similarly, *TRAIL-R2* expression is induced by p53 in response to DNA damage [84]. Therefore, DNA damage can enhance TRAIL or CD95L induced death. For example, *CD95* and *CD95L* is induced by UV in skin cells to maintain skin cell homeostasis and prevent accumulation of mutations [62].

Caspase-8 also inhibits a different form of cell death called necroptosis. Like the extrinsic apoptotic pathway, necroptosis is activated by external signals activating TNFR1. When activated at the membrane TRAILR1 forms a complex that primarily activates NFκB (Complex I). If this complex is blocked, a second cytoplasmic complex that contains receptor-interacting serine/threonine-protein kinase 1 (RIPK1) is formed, which recruits FADD and caspase-8 to induce apoptosis (Complex IIa) [4,85]. In the absence of caspase-8, RIPK1 phosphorylates, and activates receptor-interacting serine/threonine kinase 3 (RIPK3) [86]. In turn RIPK3 activates mixed lineage kinase domain like pseudokinase (MLKL), which forms pores in the plasma membrane, inducing necroptosis [87]. Components of this pathway can form a distinct complex in response to genotoxic stress called the RIPoptosome, which include RIPK1, cellular-FLICE inhibitory protein (c-FLIP), caspase-8, and FADD [88]. This complex can lead to both caspase-8-dependent apoptosis and the induction of necroptosis in response to DNA damage.

## 4. DNA Damage Induced by Caspases without Apoptosis

**Figure 3:**
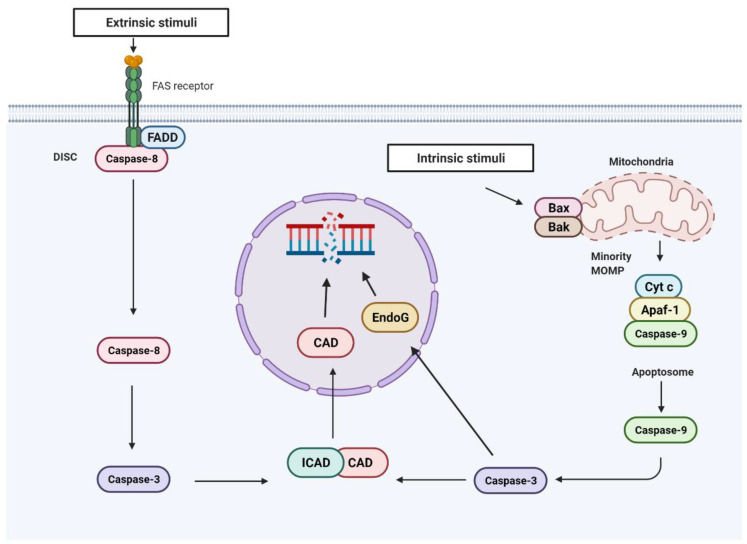
Sublethal activation of the apoptotic pathways promotes DNA damage. The intrinsic and extrinsic apoptotic pathways can be activated in the absence of cell death in the presence of sub-lethal levels of extrinsic or intrinsic stimuli. Under these conditions, caspase-8 and caspase-9 are activated by their recruitment to their activation platforms, death-inducing signaling complex (DISC), and the apoptosome, respectively. Caspase-8 and caspase-9 then activate caspase-3, which cleaves the inhibitor of caspase-activated DNAse (ICAD), resulting in the activation of caspase-activated DNAse (CAD). As an endonuclease, CAD fragments DNA, which induces DNA damage. In addition to CAD, endonuclease G (EndoG) is another downstream effector of caspase-3 that can induce DNA fragmentation under sublethal activation of the apoptotic pathways.

Apoptosis has always been considered a protecting event against genomic instability because it removes damaged cells that are in danger of becoming malignant. While MOMP has traditionally been considered an “all-or nothing” event, evidence has shown that some cells can survive the activation of this apoptotic pathway downstream of MOMP [63]. An example of this is minority MOMP, an event that results from MOMP, only occurring in a subset of the mitochondria in the cell. This leads to the sublethal activation of caspase-9, caspase-7, and caspase-3 [63]. Under these conditions caspase-3 is still capable of cleaving certain substrates, leading to DNA damage rather than apoptosis. One such substrate is the inhibitor of the caspase-activated DNAse (ICAD) (Figure 3). ICAD binds to and inhibits the activation of the caspase-activated DNAse (CAD), which is an endonuclease that cleaves DNA, producing blunt ends. During apoptosis, CAD is responsible for the characteristic DNA fragmentation that occurs [65]. When CAD is activated by the minimally active caspase-3, it leads to DNA fragmentation without apoptosis, resulting in DNA damage that accumulates, causing genomic instability and increased tumorigenic potential [63,89].

Minority MOMP has been observed to occur under different conditions that induce apoptosis, such as treatment with the BH3-mimetic ABT-737 [63] and the small molecule inhibitor of kinesin-5 (K5I) that induces prolonged mitotic arrest [89]. Importantly, it does not require the involvement of the mitochondria, because the sublethal activation of caspase-9 alone through chemical dimerization showed the same effect [63]. Interestingly, the components of the DDR engaged differed between treatments. For example, when HeLa and U2OS cells were treated with ABT-737, they did not activate the ATM or ATR pathways. Instead, there was a caspase-dependent increase in the activation of JNK1/2, showing that under these conditions c-Jun N-terminal kinases (JNK) phosphorylated H2AX [63]. γH2AX is a common indicator of the DDR as it localizes to DSB. In contrast, in G2-synchonized MCF7 cells, the increase in γH2AX observed after the treatment of cells with K5I was blocked with an ATM inhibitor but not a DNA-PK inhibitor [89]. This demonstrates that, in this context, ATM is responsible for activating the DDR in the presence of DNA damage induced by minority-MOMP. Thus, the DNA damage induced by CAD activation can engage different DNA damage pathways. These differences could be due to cell cycle status, the degree of DNA fragmentation, or cell type.

Another factor that may explain the different DNA damage pathways that are engaged by minority MOMP could be the existence of additional substrates. MDC1 is cleaved by caspase-3 during apoptosis, preventing it from interacting with ATM and thus inhibiting the DDR and preventing DNA repair [90]. While during apoptosis this inhibition of the DDR would not have any consequences, if caspase-3 can cleave MDC1 under the sublethal activation of the apoptotic pathway, this could provide another route to caspase-dependent DNA damage. Sublethal caspase-3 activation has also been associated with endonuclease G (EndoG)-induced DNA damage (Figure 3). EndoG is a nuclease that resides in the mitochondria and is from the mitochondria at the onset of MOMP and is known to induce caspase-independent DNA fragmentation [91]. The treatment of MCF10A cells with a non-lethal level of irradiation was shown to lead to caspase-3-dependent EndoG release and DNA damage. This resulted in caspase-3-dependent tumor growth [92]. As yet, it is unclear how caspase-3 promotes EndoG release or if this process is CAD-dependent. Importantly, it was not investigated whether minority MOMP occurred under these conditions.

In addition to minority MOMP, cells can also reverse apoptosis upon removal of apoptotic stimuli. Known as anastasis, this process results in genetic alterations due to DNA fragmentation, which triggers the transcription of pro-survival genes [93]. Due to the increased DNA damage and increased expression of these genes, anastasis is associated with increased oncogenesis and this could be a potential event by which cancer cells are able to accumulate novel mutations [94].

**Figure 4:**
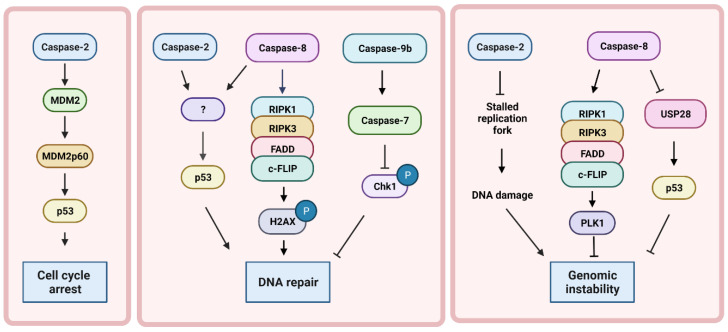
Non-apoptotic roles of caspases in the DNA damage response. Upon cytokinesis failure, caspase-2 induces p53-dependent cell cycle arrest through the cleavage of mouse double minute 2 homolog (MDM2). Under DNA damage conditions, caspase-2 regulates the activation of p53 in a MDM2-independent manner. Similarly, caspase-8 activates p53 through an unknown mechanisms and functional outcome. Caspase-8 is also involved in the phosphorylation of H2AX through the formation of a complex with receptor-interacting serine/threonine-protein kinase 1 (RIPK1), receptor-interacting serine/threonine-protein kinase 3 (RIPK3), Fas-associated protein with death domain (FADD), and the cellular-FLICE inhibitory protein (c-FLIP) known as the RIPoptosome. Unlike caspase-2 and caspase-8 that are involved in the activation of components of the DDR, the expression of a dominant negative caspase-9 (caspase-9b) results in the inhibition of Chk1 phosphorylation through the activation of caspase-7. Caspase-2 is also involved in the prevention of genomic instability by preventing stalled replication forks that could collapse and become double strand breaks. Likewise, during mitosis, the formation of the RIPoptosome results in cleavage of polo like kinase 1 (PLK1), which is crucial for the prevention of mitotic defects and genomic instability. In contrast, caspase-8 can promote the accumulation of DNA damage and genomic instability by cleaving ubiquitin specific peptidase 28 (USP28) and preventing the activation of p53.

This role of caspases in the induction of DNA damage is not exclusive to the intrinsic pathway. The treatment of mouse embryonic fibroblast (MEF) or LN18 cells with a sub-lethal dose of TRAIL was shown to activate the extrinsic pathway without inducing cell death [95,96]. Similar to minority MOMP, the activation of this apoptotic pathway led to the accumulation of DNA damage, which was dependent on caspase-8- and caspase-3-dependent cleavage of ICAD [96] (Figure 3). Thus, the sublethal activation of caspase-8 results in DNA damage in surviving cells, increasing the number of mutations leading to tumorigenesis. Caspase-8 can also prevent p53 activation through the cleavage of a deubiquitinase called ubiquitin-specific peptidase 28 (USP28), which is involved in the activation of p53 during mitotic arrest [97] (Figure 4). This allows cancer cells to overcome cell cycle arrest and to proliferate and propagate DNA damage [97]. This function of caspase-8 appear to be due to its ability to localize to the nucleus. Thus, the tumor promoting the function of caspase-8 may be regulated by its localization in the cell. Therefore, it is possible that the differential access of caspases to substrates is an important regulatory factor for caspase-induced DNA damage in the absence of cell death.

A final way in which caspases induce DNA damage is through the expression of non-apoptotic isoforms. For example, in Epstein–Barr virus (EBV)-infected primary B lymphocytes, the activation of the transcription factor STAT3 results in the upregulation of a differentially spliced variant of caspase-9 that lacks the large catalytic subunit (caspase9b) [68]. Caspase-9b acts as a dominant negative protein competing with full length caspase-9 for apoptosome binding [98]. However, the expression of caspase-9b has also been associated with the reduced phosphorylation of Chk1 [68] (Figure 4). Although non-catalytic, caspase-9b has been proposed to retain its ability to cleave caspase-7 but not caspase-3 or -6 [68]. However, this was not shown directly, but only through a peptide-based activity assay that lacks specificity [99]. However, if true, this different substrate profile may be the result of the formation of heterodimers between caspase-9b and full length caspase-9 [100]. Although the existence or enzymatic activity of these heterodimers has not been evaluated, a similar phenomenon has been observed resulting from heterodimerization of caspase-8 with its non-enzymatic homolog cFLIP [101]. Caspase-7 cleaves claspin, a Chk1 interacting protein that is required for Chk1 phosphorylation [102]. This could account for the observed reduction in that phosphorylation of Chk1. While this would be predicted to impair the DNA damage response resulting in increased DNA damage, this has not been directly shown. The alternative splicing of caspase-9 is dysregulated in non-small cell lung cancer (NSCLC), favoring the expression of caspase-9b [103]. While this would increase resistance to apoptosis, it may also lead to an impaired DNA damage response, leading to a more aggressive tumor. However, how this reduction in caspase-9b-associated Chk1 phosphorylation ultimately affects genomic instability and whether this phenomenon is specific to viral infection is yet to be determined (Table 1).

## 5. Caspase Function in DNA Repair

In addition to inducing apoptosis in response to DNA damage and inducing DNA damage itself, certain caspases have been shown to actively participate in the DDR to facilitate DNA repair (Table 1). Of these, the best characterized is caspase-2.

Loss of caspase-2 has been shown to increase genomic instability both in vivo and in vitro [104,105,106]. It may be predicted that increased genomic instability is due to the lack of apoptosis. Indeed, studies have shown that caspase-2-dependent cell death is essential in preventing aneuploidy in vitro [58,99,100]. However, the poor ability of caspase-2 to induce apoptosis in response to DNA damage argues for a separate mechanism [78]. In fact, enhancement of genomic instability in the absence of caspase-2 without affecting cell death has been demonstrated [57,106].

One potential mechanism by which caspase-2 is involved in preventing the accumulation of DNA damage independently of its apoptotic function is by regulating the cell cycle. Multiple studies have shown an increase in the proliferation of transformed cell lines in the absence of caspase-2 [68,97,98,101], and we have shown that caspase-2 is activated in dividing cells [57]. This activation occurs in G1 and appears to protect from a range of cell cycle defects. Most striking among these is the ability of caspase-2 to protect DNA replication forks. Upon the induction of replication fork stalling by treatment with hydroxyurea, loss of caspase-2 resulted in an increased percentage of stalled replication forks, new origins of replication, and delayed recovery of replication following fork restart [57]. This was accompanied by an increase in DNA damage in the absence of caspase-2 and impaired HR. Each of these observations is consistent with an impaired ATR response and bypassing of the intra-S checkpoint. Interestingly, the loss of caspase-2 did not affect Chk1 activation, suggesting that other ATR substrates may be the effectors of these functions in this context. Caspase-2 likely facilitates DNA repair through the activation of this replication checkpoint, but whether it plays an active role in DNA repair itself is still an open question.

Caspase-2 has also been shown to induce cell cycle arrest in response to polyploidy. This caspase-2 cell cycle function is dependent on p53. Upon cytokinesis failure induced through aurora B kinase inhibition in the A549 cell line, caspase-2 induced robust cleavage of the p53 negative regulator mouse double minute 2 homolog (MDM2) [58] (Figure 4). MDM2 normally binds to p53 and targets it to the proteasome for degradation. However, the fragment of MDM2 that is produced by caspase-2 can still bind to p53 but does not degrade it [59]. This results in increased p53 activity, the upregulation of p21, and cell cycle arrest. In the absence of caspase-2, the cleavage of MDM2 is blocked and p21 is not upregulated. Instead, the p53-dependent apoptotic proteins PUMA and BAX are upregulated [58]. This suggests that the caspase-2-mediated cleavage of MDM2 does not only impact p53 activation but also its choice of transcriptional targets and downstream function. The caspase-2-mediated cleavage of MDM2 appears to primarily occur when caspase-2 is activated in response to supernumerary centrioles because, in response to DNA damage or replication stress, caspase-2 does not induce MDM2 cleavage [57]. However, this does not rule out a role for p53 in caspase-2 induced cell cycle arrest that is independent of MDM2 cleavage (Figure 4). Indeed, in certain DNA damage conditions, the loss of caspase-2 attenuates p53 activation, shown by the decreased levels of p53, target gene expression [57,104]. However, as discussed above, DNA damage can activate caspase-2 in the absence of p53. In addition, increased DNA fork stalling and delayed fork restart was still observed in HeLa cells where p53 was not functional due to its inactivation through degradation by human papilloma virus E6 [57]. It is clear that there is a relationship between caspase-2 and p53 in certain conditions but the full mechanistic interplay between these two proteins and the conditions under which they function interdependently has not been fully elucidated.

This ability of caspase-2 to prevent the accumulation of DNA damage by both apoptotic and non-apoptotic functions raises the question of what determines how caspase-2 contributes to the DDR. One possible mechanism is the stage of the cell cycle. During mitosis, caspase-2-mediated apoptosis is inhibited by phosphorylation by both aurora B kinase [107] and the Cdk1/Cyclin B1 complex [108]. Aurora B kinase phosphorylates caspase-2 on position S384 [107], which blocks catalytic activity but not dimerization. S384 appears to be essential for the architecture of the catalytic site of caspase-2, allowing access to substrates [109]. In contrast, Cyclin B1 phosphorylates caspase-2 at S340 in the caspase-2 interdomain linker between the large and small catalytic subunit and this phosphorylation event blocks dimerization but not the recruitment of caspase-2 to its activation platform [108]. Phosphorylation at S340 serves to inhibit caspase-2 activation during mitosis [108]. It has been speculated that this phosphorylation event prevents caspase-2-induced apoptosis during mitosis, but it is also possible that it serves as a timer to restrict caspase-2 activation to the G1 phase of cell division. Interestingly, studies showing that caspase-2 induces apoptosis following cell cycle arrest all used inhibitors of cell cycle in G2/M, including nocodazole, mitomycin C [77], the inhibition of polo-like kinase 1 (PLK1) [66], and reversine [67]. This may suggest that caspase-2 induces different outcomes depending on the stage of the cell cycle in which it is activated, promoting DNA repair following G1 activation and apoptosis following G2 activation. Alternatively, the functions of caspase-2 may be regulated by its activation platforms. We have previously shown that under DNA damage conditions, caspase-2 is activated in the nucleolus through recruitment to a complex composed of nucleophosmin 1 (NPM1), PIDD, and RAIDD. In the presence of other cellular stressors, such as cytoskeletal disruptors, caspase-2 was activated in the cytoplasm by a different activation platform that required RAIDD but was independent of NPM1 and PIDD [78]. It is tempting to speculate that the activation of caspase-2 in different cellular compartments regulates its bifurcation of functions in DNA repair and apoptosis by giving it access to different subsets of substrates, but this has yet to be formally demonstrated.

Like caspase-2, evidence suggests that caspase-8 plays non-apoptotic roles that facilitate or directly participate in DNA repair. For example, the loss of caspase-8 leads to decreased LPS and dsRNA-induced B-cell proliferation and decreased T-cell receptor activation-induced T-cell proliferation [110,111]. Caspase-8 has been shown to impact multiple components of the DDR. In a B-cell lymphoma tumor model, the loss of caspase-8 was not only found to increase lymphomagenesis, but it also resulted in an increase in chromosomal instability [112]. This was shown to be dependent on caspase-8, as the loss of caspase-8 showed an impairment in p53 activation (Figure 4). Together, these findings show that caspase-8 plays an important role in the DNA damage response and the maintenance of chromosomal stability.

The specific activation platform for caspase-8 is also a major determinant in its ability to induce DNA damage. In hepatocytes, RIPoptosome-induced caspase-8 activation is a protector from DNA damage in hepatocytes [113]. Hepatocyte hyperproliferation induced by hepatectomy results in DNA damage specifically in replicating cells. In mice, where caspase-8 was specifically knocked out in the liver (*Casp8*^Δhep^), the number of proliferating cells positive for γH2AX was greatly reduced. A reduction in γH2AX suggests either decreased DNA damage or an impairment of the DDR upstream of γH2AX. In this case, it was the latter because pulsed-field gel electrophoresis (PFGE) confirmed the presence of DSB in *Casp8*^Δhep^ hepatocytes [113]. This is strong evidence that caspase-8 promotes DNA repair in hyperproliferation-induced replication stress. A similar effect was seen in hepatocytes treated with the DNA damage inducer doxorubicin and in hepatocytes lacking the other components of the RIPoptosome: c-FLIP, FADD, or RIPK1. This phenomenon was also independent of the apoptotic function of caspase-8 because mice expressing a catalytic mutant caspase-8 had similar levels of γH2AX as the wild-type mice [113]. This may indicate that caspase-8 functions as a scaffold protein for RIPoptosome formation in response to DNA damage (Figure 4).

The formation of the RIPoptosome was also observed during regular mitosis in HT1080, primary MEF, and HT29 cells [114]. The formation of this complex leads to the recruitment of PLK1 by RIPK1 and the cleavage of PLK1 by caspase-8 [114] (Figure 4). PLK1 regulates spindle assembly and chromosome segregation [115]. Cleavage by caspase-8 downregulates the ability of PLK1 to interact with and phosphorylate its substrate BUBR1 [114]. BUBR1 prevents transition from metaphase to anaphase until the chromosomes are properly aligned [116]. However, both blocking BUBR1 phosphorylation and hyperactive BUBR1 result in the misalignment of chromosomes during mitosis, which can lead to mitotic defects. Therefore, the cleavage of PLK1 by caspase-8 likely serves to modulate BUBR1 activity levels to ensure correct cell division and chromosomal separation. Consistent with this, the enhancement of RIPoptosome formation by the inhibitor of apoptosis (IAP) inhibition and disruption of the complex by the knockout of RIPK1 or caspase-8 both resulted in mitotic defects. Thus, a balanced assembly of this complex to ensure correct cleavage of PLK1 is essential for proper chromosome segregation and genomic stability [114].

## 6. Crosstalk between Inflammation, Caspases, and DNA Damage

The caspase family also contains of a third group known as the inflammatory caspases. This group is comprised of caspase-1, caspase-4, and caspase-5 in humans and caspase-11 in mice. Unlike the apoptotic caspases, these caspases are involved in the induction of a different type of cell death known as pyroptosis. Pyroptosis is an inflammatory type of cell death generally induced by damage-associated molecular patterns (DAMPs) and pathogen-associated molecular patterns (PAMPs) that trigger the formation of complexes known as inflammasomes, which activate caspase-1 [117]. Once activated, caspase-1 is responsible for the cleavage of substrates, such as gasdermin D (GSDMD) and pro-IL-1β, that culminate in cell death. Alternatively, stimuli, such as lipopolysaccharide (LPS), can activate caspase-4, caspase-5, and caspase-11, which can also cleave GSDMD and result in pyroptosis [117].

Inflammasomes have also been shown to respond to DNA-damaging stimuli like radiation [118,119]. Ionizing radiation induces cell death and tissue damage in radiosensitive cells like the intestine and hematopoietic cells. This has been shown to be dependent on caspase-1 activated by the AIM2 inflammasome, which is activated by damaged DNA in the nucleus [118]. The activation of inflammatory caspases without the induction of cell death has also been observed in response to a variety of stimuli including oxidized lipids (oxPAC) [120], bacterial peptidoglycan [121], and LPS [122,123]. This phenomenon is known as hyperactivation and results release in IL-1β through GSDMD pores while maintaining cell viability [124]. Whether DNA damage can similarly engage this process has not been explored. Interestingly, in a mouse model of inflammation-induced colon cancer, the loss of caspase-1 or NLRC4 enhanced tumorigenesis [56]. This was not due to an effect on inflammation but rather increased cell proliferation and reduced apoptosis. Thus, in this cancer model, caspase-1 and NLRC4 have non-inflammatory anti-proliferative effects, potentially decreasing mutagenesis and genomic instability.

Crosstalk between the apoptotic and inflammatory pathways has been demonstrated. For example, in the absence of caspase-1, caspase-3 can cleave GSDME to induce pyroptosis [125]. GSDME can activate caspase-3 in response to UV treatment by permeabilizing the mitochondria [54]. Finally, caspase-1 can induce caspase-3 activation and apoptosis in the absence of GSDMD [53,55]. When caspases are inhibited permeabilization of the inner mitochondrial membrane results in release of mtDNA. This leads to the activation of the cGAS/STING pathways and the upregulation of interferon [126]. cGAS has been shown to inhibit HR by binding to PARP1 at DSBs and inhibiting its downstream function [127]. Thus, in a scenario of sublethal caspase activation, caspases can protect DNA repair pathways by inhibiting a proinflammatory pathway.

## 7. Caspases in Cancer

One of the hallmarks of cancer is the evasion of apoptosis [128]. Due to this, members of the caspase family have always been considered the protectors of tumor formation (Table 2). Indeed, caspase-2 functions as a tumor suppressor in a number of mouse models driven by different oncogenic activators in both hematological malignancies [80,106] and solid tumors [105,129]. In human cancer, the low expression of caspase-2 correlates with worse prognosis in acute myeloid leukemia [130]. In addition, somatic mutations in the *CASP2* gene have been found in gastric and colorectal cancers [131]. Similarly, the low expression of caspase-8 in neuroblastoma with N-MYC amplification and small cell lung carcinoma and somatic mutations in *CASP8* hepatocellular carcinoma are all associated with increased tumorigenesis [132,133,134]. Interestingly, both caspase-2 and caspase-8 have been shown to play a tumor-promoting role. In a mouse TH-MYC neuroblastoma tumor model, the loss of caspase-2 was found to delay tumorigenesis [135]. Likewise, in human cancer, the high expression of *CASP8* promotes tumorigenesis in breast and pancreatic cancers [136]. Although the role of caspase-3 as a tumor suppressor has been less well established, a study of breast cancer samples showed a lack of caspase-3 expression in 75% of the samples [137]. However, in another study, caspase-3 was found to be upregulated in breast cancer tumors [138]. While it seems contradictory that proteins that are usually involved in the removal of malignant cells could both promote and suppress tumorigenesis, the non-apoptotic roles of caspases in the accumulation of genomic instability likely account for these opposing functions. For example, in certain cells, caspase-8 engages the intrinsic pathway through BID cleavage [139]. It is possible that the high expression of caspase-8 in tumors results in a low level of residual caspase-8 activation that cleaves enough BID to induce minority MOMP and DNA damage, leading to tumorigenesis, while full caspase-8 activation would lead to apoptosis and tumor cell removal. Thus, the mechanisms and functional outcomes of caspase activation in response to DNA damage are likely intrinsically dependent on the extent of the damage.

## 8. Closing Remarks

Since their discovery, the members of the caspase family have been known for their roles in inducing cell death. However, increased evidence shows that they play other important roles independent of their cell death function to induce, prevent, and repair DNA damage. The roles of caspases in the DDR enlightens the mechanisms by which different caspases can function to both suppress and promote tumor growth. A further understanding of the non-apoptotic roles of caspases is crucial as the activation of caspases is commonly used for cancer treatment. In some cases, instead of inhibiting tumor growth, caspase activation has the potential to promote tumor growth and enhance resistance. For example, there is a lot of focus on the potential of using BH3-mimetics, FLIP inhibitors, and extrinsic ligands like TRAIL for the elimination of cancer cells [4]. In fact, the BCL2-targeting BH3 mimetic ABT-199 (Venetoclax) is the only drug that directly targets the apoptotic pathway which is FDA approved for cancer treatment [140]. While the success of this drug and the others in development is highly encouraging, their use in the clinic underscores how important it is to understand the full spectrum of consequences of caspase activation with respect to the induction or prevention of DNA damage.

## Figures and Tables

**Table 1:** Functions of caspases in the DDR.

Caspase	Lethal Role	Non-Lethal Role
Caspase-1/4/5/11	GSDMD and pro-IL-1β cleavage.	Potential sublethal activation of caspase-3 [53,54,55]; suppression of proliferation in colorectal cancer [56]
Caspase-2	Bid cleavage; intrinsic pathway activation	DNA replication fork protection; ploidy-induced cell cycle arrest; MDM2 cleavage [57,58,59].
Caspase-3	Execution of apoptosis	ICAD cleavage; DNA damage; EndoG release [60,61,62,63]; inhibition of cGAS/STING [64]
Caspase-8	Extrinsic pathway initiation; caspase-3 and -7 activation; Bid cleavage	Cleavage of USP28; RIPoptosome formation; PLK1 cleavage [65,66,67]
Caspase-9	Intrinsic pathway initiation; caspase-3 and -7 activation.	Reduced Chk1 activation by caspase-9b [68]

**Table 2:** Summary of the role of caspases in cancer.

Caspase	Role in Human Cancers
Caspase-1	Tumor suppressor in a colon cancer model [56]
Caspase-2	Tumor suppressor in hematological malignancies and epithelial cancers [80,106]; promotes tumorigenesis in TH-MYC neuroblastoma [135].
Caspase-3	Promotion and prevention of tumorigenesis in breast cancer [137,138].
Caspase-8	Low expression in hepatocellular carcinomas, small cell lung carcinoma, and neuroblastomas with N-MYC amplification associated with tumorigenesis [132,133,134]; high expression in breast and pancreatic cancer associated with tumorigenesis [136].

## Data Availability

Not applicable.
